# Exotic invaders gain foraging benefits by shoaling with native fish

**DOI:** 10.1098/rsos.140101

**Published:** 2014-11-19

**Authors:** Morelia Camacho-Cervantes, Constantino Macías Garcia, Alfredo F. Ojanguren, Anne E. Magurran

**Affiliations:** 1 Centre for Biological Diversity, University of St Andrews, Sir Harold Mitchell Building, St Andrews, UK; 2 Instituto de Ecología, Universidad Nacional Autónoma de México, Ciudad Universitaria, México D F, Mexico

**Keywords:** interspecific interactions, invasion success, guppies, goodeids, sociability, Allee effects

## Abstract

Freshwater habitats are under increasing threat due to invasions of exotic fish. These invasions typically begin with the introduction of small numbers of individuals unfamiliar with the new habitat. One way in which the invaders might overcome this disadvantage is by associating with native taxa occupying a similar ecological niche. Here we used guppies (*Poecilia reticulata*) from a feral population in Mexico to test the prediction that exotic shoaling fish can associate with heterospecifics, and that they improve their foraging efficiency by doing so. Guppies have invaded the Mexican High Plateau and are implicated in the declines of many native topminnow (Goodeinae) species. We show that heterospecific associations between guppies and topminnows can deliver the same foraging benefits as conspecific shoals, and that variation in foraging gains is linked to differences in association tendency. These results uncover a mechanism enabling founding individuals to survive during the most vulnerable phase of an invasion and help explain why guppies have established viable populations in many parts of Mexico as well in every continent except Antarctica.

## 2. Introduction

Invasive species, a major agent of global change [1,2], modify the environment at multiple ecological levels, lead to community disassembly and alter species interactions across a range of spatial and temporal scales [2–4]. These changes result in biodiversity loss and wildlife homogenization [5] and are considered some of the greatest threats to ecosystem services [6,7].

Although many species are translocated from their native range, most do not establish viable populations [1,2]. Invasions typically begin with the introduction of just a few individuals [1], and behaviour may play a crucial role in enabling such individuals to compensate for Allee effects—the disadvantages linked to membership of a small population [8,9]—and to survive until they can reproduce [10,11].

In fish, as in other taxa, social behaviour can enhance survival [12]. Apart from for mating, fish associate with other individuals in contexts such as hibernation, sleeping and foraging [13], thus gaining benefits including protection from predators [14], increased foraging efficiency [15] and reductions in the energetic costs of movement [12]. However, animal associations are not limited to single species groups. Mixed-species (heterospecific) aggregations, i.e. two or more species associating in time and space [16], occur regularly in nature; examples include fish [17,18], birds [19] and even members of very distant taxa (e.g. monkeys and birds [20]). Heterospecific aggregations occur when they are beneficial to the participants [21]. For example, fathead minnows (*Pimephales promelas*) can learn to recognize heterospecific alarm cues, and this decreases their probability of being attacked and captured during predator encounters [22].

Freshwater ecosystems are among the most altered and invaded in the world [23]. Like islands, they are vulnerable due to their geographical isolation and high rates of endemicity [24]. Common routes of fish invasion include introductions of biological control agents [25], releases designed to provide food and sport or discards of aquarium fish and bait buckets [26]. Although in some cases freshwater fish invasions may have a positive outcome for the local fish communities and on human economy [27], in others their effects are catastrophic [28]. Freshwater invaders are responsible for effects that range from local extinctions to alterations in nutrient and energy fluxes [29].

The guppy (*Poecilia reticulata*) is native to Trinidad, Guyana, Venezuela and Surinam [30,31]. It is a remarkably opportunistic species with reproductive adaptations that enable a few individuals or even a single pregnant female to found a viable population [31]. Guppies possess many of the physiological, behavioural and life-history characters that are associated with extreme adaptability [31]—traits associated with increased invasion success [32]. During the past century, guppies have been released into environments outside their native range to control mosquitoes and reduce malaria, and also accidentally as a consequence of escapes from home aquaria. There are now established populations in at least 72 different countries across the globe [33]. This includes Mexico [34], where they are found in many localities including the Lerma-Santiago River system, the main basin of the Mexican High Plateau and a watershed noted for its high levels of endemicity. Endemics include Goodeinae, a clade consisting of *ca* 45 species of small livebearing fish [35], 17 of which are included in the IUCN Red List of Threatened Species [36] (see also [37,38]). The Goodeinae are mostly omnivorous freshwater topminnows that inhabit shallow ponds, lakes and rivers. They are the focus of this study because many species are morphologically similar to guppies, feed on the same resources and occupy the same habitat. In some cases, population declines have been directly attributed to guppy invasions [39].

**Figure 1.**
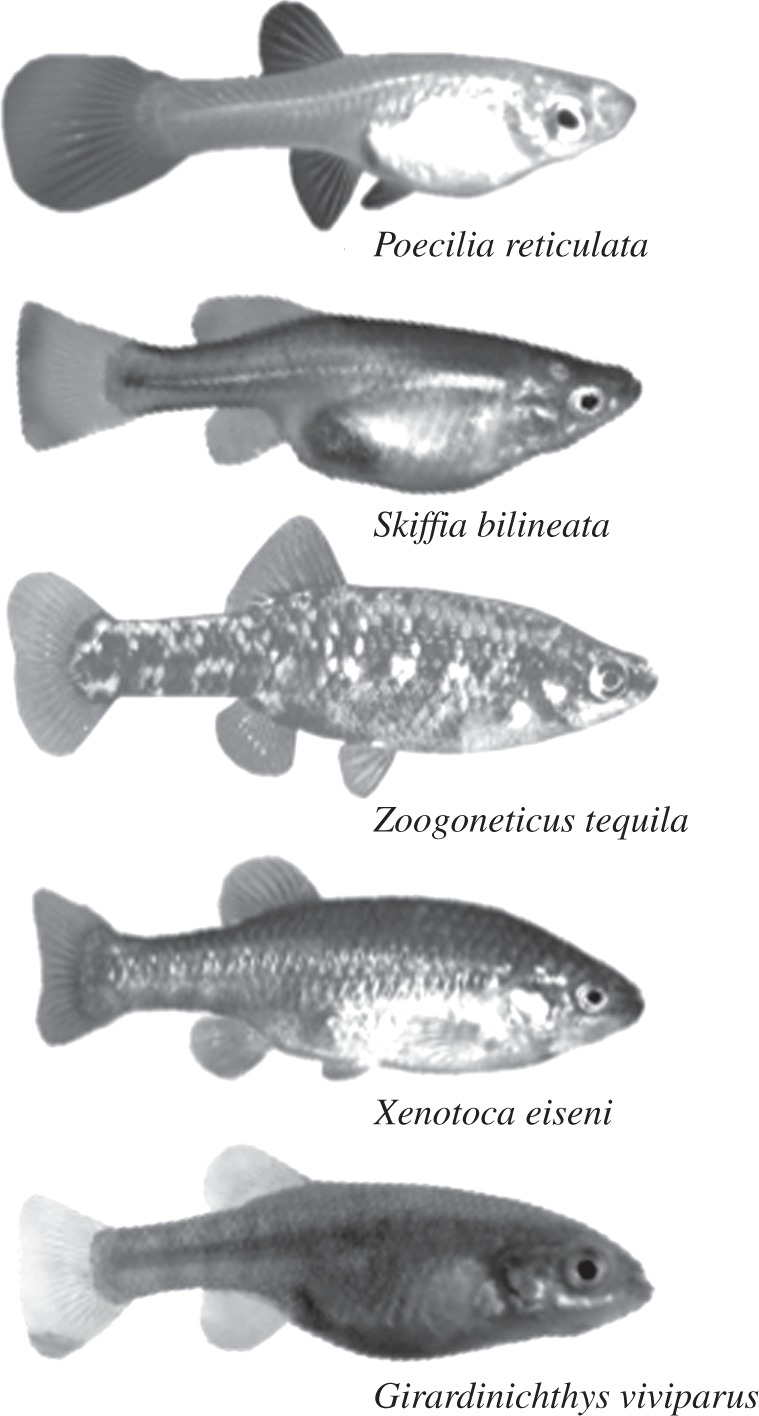
Species used in these experiments, all individuals are adult females (photo composition by the authors).

We tested the hypothesis that small shoals of invading guppies gain foraging benefits by associating with topminnows. We quantified foraging benefits associated with an increase in conspecific shoal size. We expected that fish would locate food faster and increase the time spent foraging when associating with others [40]. We predicted that foraging advantages would also apply when the additional shoal members were heterospecific rather than conspecific fish. To assess whether these effects can be generalized across species we repeated the experiments with four topminnow species (*Skiffia bilineata*, *Zoogoneticus tequila*, *Xenotoca eiseni* and *Girardinichthys viviparous*; figure 1) that are morphologically similar to guppies [41]. In addition, we asked whether the differences in the foraging advantages that accrue when individuals belong to a larger shoal could be linked to the guppy's tendency to associate with a given species.

## 3. Material and methods

Experiments were carried out at the main campus of the National Autonomous University of México (UNAM) in México City from July to September 2013. Guppy (*P. reticulata)* individuals were collected from a population established in the wild in Ahuisculco, Jalisco, where no other species used in this experiment occur. In the case of the topminnows, *Z. tequila* were originally from Teuchitlán in Jalisco; *G. viviparus* originated in Texcoco, México; *S. bilineata* were originally from Álvaro Obregón in Michoacán and *X. eiseni* from San Sebastián in Jalisco. All fish were collected from either the wild or outdoor ponds within a two-week period, and carefully transported in plastic bags half filled with water and half filled with air to the laboratory, where they remained for roughly the same amount of time (*ca* 12 days) before trials. Stock tanks (45 l) contained 15–20 fish each and were set up with aged tap water, which was treated with Stress Coat. Each tank contained a filter, water pump and plants. Photoperiod was 12 L : 12 D from 7.00 to 19.00 h. Water daily temperature ranged between 19°C and 22°C. Tanks were visually isolated from one another with an opaque sheet. We used only female fish in the experiment as they devote more time to shoaling and foraging than males [42]. Individuals in a given trial were kept separate for several weeks prior to observations to avoid familiarity effects [43]. Fish were fed with commercial flake food (SeraVipan) daily at the end of each day. After the experiment was completed (70 days), all fish remained in stock tanks in the laboratory. In the wild, species used in this study have similar foraging patterns and forage from similar sources: plants, detritus and smaller animals [35]. Nevertheless, *Z. tequila* is, among the species used in these experiments, the most likely to feed at the bottom [44].

Our study was divided into two parts: in the first we measured foraging behaviour in the presence of mixed or single species shoals (*foraging benefits test*). We then evaluated whether guppies would shoal with topminnows (*heterospecific association test*). In the two parts, we selected a guppy prior to the start of each observation (focal) and recorded its behaviour; they were easily distinguished from the rest of the fish due to minor individual differences, such as eye size or fin scars. Focals were used only once and returned to stock tanks after each trial. Fish used to form the shoals were haphazardly selected from three tanks holding approximately 15 fish of one species each to avoid pseudoreplication [45]. Observations were made between 10.00 and 16.00 h using two identical glass tanks (45×25×30 cm) each with a gravel bottom.

In the *foraging* trials, pelleted fish food (Pleco Sticks) was placed at the bottom of a randomly selected corner of the tank at the beginning of the day. Shoals were assembled with a female guppy from the focal tanks and haphazardly selected individuals from the shoal tanks to produce the desired composition for a given trial, then gently introduced to the observation tank. Shoals typically consisted of three guppy females and three females of one Goodeinae species. We also included two conspecific shoal sizes (of three or six guppies) to assess whether a change in food finding linked to an increase in a single species group size is matched when 50% of the conspecific individuals are replaced by heterospecifics. The shoal was observed for 10 min to determine both the time (seconds) and species of the first fish to locate the food. We recorded the time (seconds) it took the first fish to locate the food and also the time (seconds) it took the focal guppy to do it. We then recorded the time spent foraging by the focal guppy female during the rest of the trial. As some individuals had more time left than others, data for this variable were analysed using the proportion of time spent foraging from the available time (time spent foraging divided by the remaining time after the food was located). Each of the six treatments was replicated 22 times. Replicates for all treatments were performed in a random order.

For the *heterospecific* *association* trials, all shoals consisted of six fish (in one treatment these were all guppies, in the others the shoal consisted of three guppies and three Goodeinae of the same species). Shoals were assembled as before and then gently placed in a bottomless bottle inside the observation tank to acclimatize for 10 min; the bottle was then carefully lifted and removed. The focal female was then followed for 8 min. Every 15 s we recorded the species and distance (spot sampling), in body lengths, to the closest heterospecific and conspecific fish. Each of the five treatments was replicated 15 times in a random order.

Standard length of the fish used in these experiments ranged from 17.2 to 35.1 mm. However, the shoals and focals were size assorted trying to minimize differences in size that could influence behaviour. Average (±s.d.) difference between the standard length (SL) of the focal and the average SL of the shoal fish (i.e. relative size of the focal fish) was −0.6±1.5 mm (ranging from −4.1 to 3.2 mm) and was not significantly different across treatments of shoal composition (ANOVA, *F*_5,126_=0.72, *p*=0.61). However, all analyses were performed including difference in size as a covariate. Since neither difference in size (*F*<0.783, *p*>0.39) nor the interaction between difference in size and treatment (*F*<0.465, *p*>0.5) had a significant effect, we concluded that size did not play a role in foraging or association patterns in this experiment. Therefore, for the benefit of clarity, the Results section only presents the analyses with treatment as the main factor.

In the Foraging benefits section, in order to evaluate the foraging benefits obtained by guppies in shoals of different compositions we first asked (using *χ*^2^-tests) whether one species in the two species trials consistently found the food first. Next we examined the time taken by the focal female to begin foraging. These data were log transformed to approximate normality. An ANOVA, followed by Tukey HSD post-hoc tests was then used to assess the differences among treatments.

In the Heterospecific association section, we examined association patterns using ANOVA. In the first test, we asked whether the number of occasions in a trial (out of a maximum of 32) on which the focal female was shoaling with a conspecific, defined as the focal female being within one body length of another guppy, varied between treatments. In the second test, we asked whether the extent to which focal females shoaled with heterospecifics, defined using the one body length criterion as before, depended on the species of topminnow involved. Post-hoc Tukey tests were used when treatment effects were significant. All analyses were performed using R statistical software [46].

## 4. Results

### 4.1 Foraging benefits

**Figure 2.**
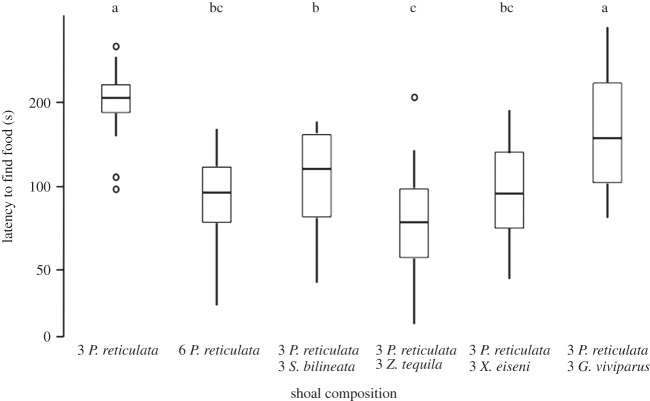
Time (maximum=600 s, in a log scale) the guppy focal fish took to find the food for each shoal composition. Horizontal lines in the bars represent the median, boxes indicate interquartile ranges and vertical lines show the range excluding outliers (circles). Letters represent the results of a Tukey HSD post-hoc test.

**Figure 3.**
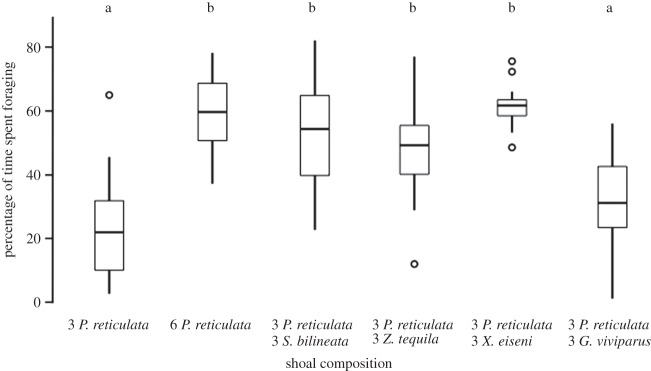
Percentage of the time after finding food that the focal spent eating for each shoal composition. Horizontal lines in the bars represent the median, boxes indicate interquartile ranges and vertical lines show the range excluding outliers (circles). Letters represent the results of a Tukey HSD post-hoc test.

The time taken for the focal fish to find food varied across treatments (*F*_5,75_=20.39, *p*<0.001; figure 2). Post-hoc tests revealed that when guppies were in a single species shoal of six, the focal female found food more quickly than when there were three guppies in the tank. This advantage also occurred in three out of the four cases when the shoal was composed of both guppies and topminnows (i.e. in the presence of *S. bilineata*, *Z. tequila* or *X. eiseni* but not when the additional fish were *G. viviparus*). Focal individuals also increased the proportion of time they spent foraging when the shoal increased from three to six in all treatments, except—again—in the case of *G. viviparus*, where the focal female behaviour was indistinguishable from that exhibited in a shoal of three guppies (*F*_5,75_=26.65, *p*<0.001; figure 3). With the exception of the trials with *G. viviparus*, the heterospecific shoal members located the hidden food more quickly, or as quickly as shoal with only guppies did (table 1).

**Table 1.** Species of the first fish to locate the food in the 22 replicates of the trials to evaluate *foraging benefits*; *p*-values from *χ*^2^-tests. All treatments included three individuals of each species.

treatment	guppies first	heterospecific first	*p*-value
*P. reticulata*	6	16	0.033
*S. bilineata*			
*P. reticulata*	1	21	<0.001
*Z. tequila*			
*P. reticulata*	9	13	0.393
*X. eiseni*			
*P. reticulata*	17	5	0.010
*G. viviparus*			

### 4.2 Heterospecific association

**Figure 4.**
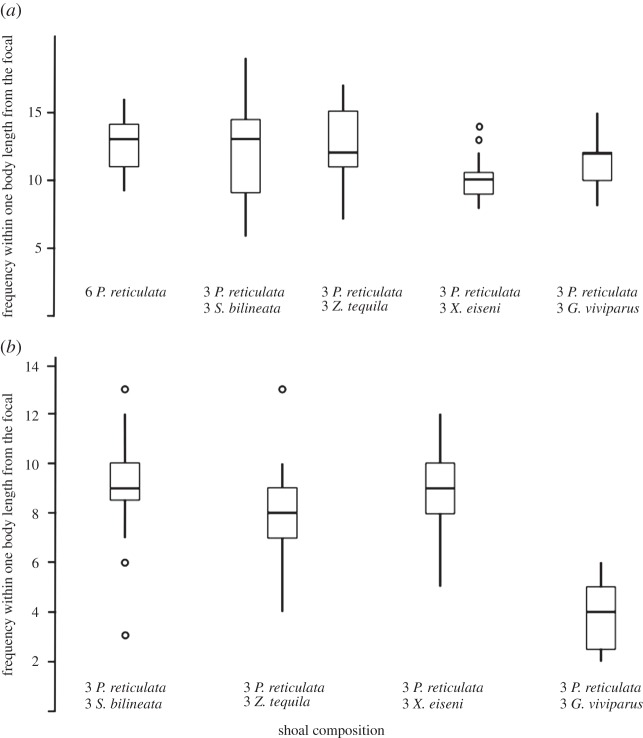
Times (maximum=32) focal fish were found within one body length or less from the (*a*) closest conspecific and (*b*) heterospecific. Horizontal lines in the bars represent the median, boxes indicate interquartile ranges and vertical lines show the range excluding outliers (circles).

The number of times the closest guppy was found within one body length of the focal female was not significantly different in all treatments (*F*_4,75_=2.25, *p*=0.071). However, the extent to which the focal females shoaled with heterospecifics varied between treatments (*F*_3,60_=23.49, *p*<0.001; figure 4). Post-hoc tests revealed that guppies were less likely to associate with *G. viviparous* than with any of the other three species of Goodeinae, but equally likely to associate with the latter three species as with conspecifics.

## 5. Discussion

Our data demonstrate that guppies—regarded as one of the world's most invasive freshwater fish—gain the same benefits, in terms of finding hidden food sources, when shoaling with native heterospecifics as they would by belonging to a conspecific shoal of the same size. Being part of a large shoal of conspecifics enhances foraging success of the individuals that constitute it [40]. Guppies are among the species in which it has been shown that social interactions can result in foraging benefits [15,47,48]. Individuals lacking information about the local environment can, if joining a group, learn from other more knowledgeable conspecifics [49]. Indeed, foraging information may be transmitted by processes as simple as the tendency to follow other fish [47]. Here we have shown that these benefits extend across, as well as within, species.

In our trials, topminnows were often the first to find the food, with guppies subsequently locating it. We refer to Goodeinae fish as topminnows because they, as the guppies, regularly forage at the water surface, yet they also forage at the bottom, and it has been reported that *Zoogoneticus* spp. are more likely to forage from the substrate than other Goodeid genera [44]. Thus, it is possible that our protocol made topminnows more likely to find the food pellets than the guppies. If so, the fact that female guppies were better able to find and consume pellets at the bottom when shoaling with topminnows is evidence that their behaviour is flexible enough to allow them to benefit from shoaling with native species. It must be noted, however, that guppy females are also likely to forage at the bottom under some circumstances [31], which may explain why they were also able to locate food faster and spend more time foraging when in larger shoals of conspecifics.

Being able to follow other individuals to find food more efficiently would annul one major disadvantage that locally scarce invading fish have to face [8]. Yet there are advantages of belonging to a larger group other than faster location of hidden food. A major benefit of these associations is the increased vigilance associated with ‘many eyes’ [50]. It is believed that there is a positive relationship between being a successful forager and avoiding predators [51]. Larger flocks or shoals are better at detecting approaching predators and taking advantage of the dilution effect, but, crucially, the individual members devote less time to scanning for potential threats [51,52]. This effect, which leaves more time for feeding, occurs even in the absence of an evident predation risk and helps reduce the individual fitness cost of predation [53].

In our investigation, the focal females not only found food faster in the larger shoals (whether the additional shoal members were conspecifics or heterospecifics) but devoted more time to foraging. Indeed, the link between the tendency to associate with a given topminnow species and the foraging advantages that accrue when it is present, directly implies shoaling behaviour as a cause of the foraging gains. In short, our results substantially extend earlier research on single species shoals by showing that the foraging advantages of increased shoal size apply when the additional conspecifics are replaced by heterospecifics. However, as our experimental design included only females, further research should be carried on to explore whether these advantages remain when guppy and Goodeinae males are part of the group. Indeed, it is known that guppy males interact with native Mexican topminnows and even attempt to copulate with them [41].

While Poeciliids, including the guppy, possess many of the traits associated with successful invaders [31,33,54,55] such as phenotypic plasticity [56,57], ovoviviparity [31] and a flexible life history [58] the likelihood that founders will establish a viable population may depend on many local factors including the traits of the species that already occur there. There is no consensus regarding which species or community attributes promote invader success or explain spread dynamics [5,59]. Among freshwater fish invasions, establishment success is the most studied phase and it seems to be multi-factorial and dependent on the context. For example in the USA, 87 species of fish are known to have been introduced to California, and among these the main predictors of establishment success are physiological tolerance, smaller size of native range and—somewhat circularly—prior invasion success [59].

The natural habitat of most of the topminnow species used in this study has already been invaded to a lesser or greater extent—and often intermittently—by guppies. It is therefore likely that invading guppies in Mexico have already been able to exploit the foraging and other benefits of heterospecific shoaling. Indeed, in the site were we collected guppies for this study (Ahuisculco, Jalisco) they were in close association with other species, as inferred from the fact that we found more than one species in our nets. However, a further important finding of our work is that not all native species that might be encountered will deliver the same foraging gains. Indeed, in our study associations with *G. viviparus* brought no foraging gains. This outcome highlights the context-dependent nature of invasions [5].

The number of species that successfully establish themselves outside their native range is increasing, as is the number of these that cause economic and ecological damage [10,60]. Our results suggest that plastic social behaviour could help invading species to overcome initial numerical disadvantages and become successful invaders. This, together with direct negative effects on local species (e.g. introduction of novel parasites and sexual disruption), may facilitate the establishment of viable populations and the eventual replacement of native species. This study reveals that sociability is one of the key predictors of species establishment in novel localities. It highlights the need to pay attention to behavioural traits when assessing the invasion risk associated with releases or escapes of exotic species.

## Supplementary Material

results of the association section are in the “association.Nov18.txt”

## Supplementary Material

results of the foraging section are in “foragingMx2013.prop.txt”

## Data accessibility

Data can be found in the electronic supplementary material.

## References

[1] Mack RN, Simberloff D, Lonsdale WM, Evans H, Clout M, Bazzaz FA 2000 Biotic invasions: causes, epidemiology, global consequences, and control. *Ecol. Appl.* 10, 689–710. (doi:10.2307/2641039)

[2] Lockwood JL, Hoopes MF, Marchetti MP 2006 *Invasion ecology.* Oxford, UK: Blackwell Publishing.

[3] Sanders NJ, Gotelli NJ, Heller NE, Gordon DM 2003 Community disassembly by an invasive species. *Proc. Natl Acad. Sci. USA* 100, 2474–2477. (doi:10.1073/pnas.0437913100) 12604772 10.1073/pnas.0437913100 PMC151365

[4] Ehrenfeld JG 2010 Ecosystem consequences of biological invasions. *Annu. Rev. Ecol. System.* 41, 59–80. (doi:10.1146/annurev-ecolsys-102209-144650)

[5] Arim M, Abades SR, Neill PE, Lima M, Marquet PA 2006 Spread dynamics of invasive species. *Proc. Natl Acad. Sci. USA* 103, 374–378. (doi:10.1073/pnas.0504272102) 16387862 10.1073/pnas.0504272102 PMC1326152

[6] Pejchar L, Mooney HA 2009 Invasive species, ecosystem services and human well-being. *Trends Ecol. Evol.* 24, 497–504. (doi:10.1016/j.tree.2009.03.016) 19577817 10.1016/j.tree.2009.03.016

[7] Wilcove DS, Rothstein D, Dubow J, Phillips A, Losos E 1998 Quantifying threats to imperiled species in the United States. *Bioscience* 48, 607–615. (doi:10.2307/1313420)

[8] Tobin PC, Berec L, Liebhold AM 2011 Exploiting Allee effects for managing biological invasions. *Ecol. Lett.* 14, 615–624. (doi:10.1111/j.1461-0248.2011.01614.x) 21418493 10.1111/j.1461-0248.2011.01614.x

[9] Courchamp F, Berek L, Gascoigne J 2008 *Allee effects in ecology and conservation.* Oxford, UK: Oxford University Press.

[10] Holway DA, Suarez AV 1999 Animal behavior: an essential component of invasion biology. *Trends Ecol. Evol.* 14, 328–330. (doi:10.1016/S0169-5347(99)01636-5) 10407433 10.1016/s0169-5347(99)01636-5

[11] Stephens PA, Sutherland WJ 1999 Consequences of the Allee effect for behaviour, ecology and conservation. *Trends Ecol. Evol.* 14, 401–405. (doi:10.1016/s0169-5347(99)01684-5) 10481204 10.1016/s0169-5347(99)01684-5

[12] Krause J, Ruxton GD 2002 *Living in groups.* New York, NY: Oxford University Press.

[13] Bleakley BH, Parker DJ, Brodie ED III 2007 Nonadditive effects of group membership can lead to additive group phenotypes for anti-predator behaviour of guppies,?. *Poecilia reticulata.* 20, 1375–1384. (doi:10.1111/j.1420-9101.2007.01342.x) 10.1111/j.1420-9101.2007.01342.x 17584232

[14] Magurran AE, Nowak MA 1991 Another battle of the sexes: the consequences of sexual asymmetry in mating costs and predation risk in the guppy, *Poecilia reticulata. Proc. R. Soc. Lond. B* 246, 31–38. (doi:10.1098/rspb.1991.0121) 10.1098/rspb.1991.0121 1684666

[15] Day RL, MacDonald T, Brown C, Laland KN, Reader SM 2001 Interactions between shoal size and conformity in guppy social foraging. *Anim. Behav.* 62, 917–925. (doi:10.1006/anbe.2001.1820)

[16] Morse DH 1970 Ecological aspects of some mixed-species foraging flocks of birds. *Ecol. Monogr.* 40, 1–119. (doi:10.2307/1942443)

[17] Sazima C, Krajewski JP, Bonaldo RM, Sazima I 2007 Nuclear-follower foraging associations of reef fishes and other animals at an oceanic archipelago. *Environ. Biol. Fish.* 80, 351–361. (doi:10.1007/s10641-006-9123-3)

[18] Camacho-Cervantes M, Ojanguren AF, Deacon AE, Ramnarine IW, Maguran AE 2013 Association tendency and preference for heterospecifics in an invasive species. *Behaviour* 151, 769–780. (doi:10.1163/1568539X-00003169)

[19] Farine DR, Milburn PJ 2013 Social organisation of thornbill-dominated mixed-species flocks using social network analysis. *Behav. Ecol. Sociobiol.* 67, 321–330. (doi:10.1007/s00265-012-1452-y)

[20] Boinski S, Scott PE 1988 Association of birds with monkeys in Costa Rica. *Biotropica* 20, 136–143. (doi:10.2307/2388186)

[21] Ward AJW, Axford S, Krause J 2002 Mixed-species shoaling in fish: the sensory mechanisms and costs of shoal choice. *Behav. Ecol. Sociobiol.* 52, 182–187. (doi:10.1007/s00265-002-0505-z)

[22] Chivers DP, Mirza RS, Johnston JG 2002 Learned recognition of heterospecific alarm cues enhances survival during encounters with predators. *Behaviour* 139, 929–938. (doi:10.1163/156853902320387909)

[23] Garcia-Berthou E, Alcaraz C, Pou-Rovira Q, Zamora L, Coenders G, Feo C 2005 Introduction pathways and establishment rates of invasive aquatic species in Europe. *Can. J. Fish. Aqu. Sci.* 62, 453–463. (doi:10.1139/f05-017)

[24] Moyle PB 1996 *Effects of invading species on freshwater and estuarine ecosystems* (eds Sandlund OT, Schei PJ, Viken A), pp. 86–92. Trondheim, Norway: Norwegian Institute of Nature Research (NINA).

[25] Englund RA 1999 The impacts of introduced poeciliid fish and Odonata on the endemic Megalagrion (Odonata) damselflies of Oahu Island, Hawaii. *J. Insect Conserv.* 3, 225–243. (doi:10.1023/a:1009651922486)

[26] Strayer DL 2010 Alien species in fresh waters: ecological effects, interactions with other stressors, and prospects for the future. *Freshwater Biol.* 55, 152–174. (doi:10.1111/j.1365-2427.2009.02380.x)

[27] Gozlan RE 2008 Introduction of non-native freshwater fish: is it all bad??. *Fish Fish.* 9, 106–115. (doi:10.1111/j.1467-2979.2007.00267.x)

[28] Vitule JRS, Freire CA, Simberloff D 2009 Introduction of non-native freshwater fish can certainly be bad. *Fish Fish.* 10, 98–108. (doi:10.1111/j.1467-2979.2008.00312.x)

[29] Simon KS, Townsend CR 2003 Impacts of freshwater invaders at different levels of ecological organisation, with emphasis on salmonids and ecosystem consequences. *Freshwater Biol.* 48, 982–994. (doi:10.1046/j.1365-2427.2003.01069.x)

[30] Magurran AE, Seghers BH, Shaw PW, Carvalho GR 1995 The behavioral diversity and evolution of guppy, Poecilia reticulata, populations in Trinidad. *Adv. Study Behav.* 24, 155–202. (doi:10.1016/S0065-3454(08)60394-0)

[31] Magurran AE 2005 *Evolutionary ecology: the Trinidadian guppy.* Oxford, UK: Oxford University Press.

[32] Hellmann JJ, Byers JE, Bierwagen BG, Dukes JS 2008 Five potential consequences of climate change for invasive species. *Conserv. Biol.* 22, 534–543. (doi:10.1111/j.1523-1739.2008.00951.x) 18577082 10.1111/j.1523-1739.2008.00951.x

[33] Deacon AE, Ramnarine IW, Magurran AE 2011 How reproductive ecology contributes to the spread of a globally invasive fish. *PLoS ONE* 6, 24416 (doi:10.1371/journal.pone.0024416) 10.1371/journal.pone.0024416 PMC3176282 21957449

[34] Contreras-MacBeath T, Mejia Mojica H, Carrillo Wilson R 1998 Negative impact on the aquatic ecosystems of the state of Morelos, Mexico from introduced aquarium and other commercial fish. *Aquarium Sci. Conserv.* 2, 67–78. (doi:10.1023/A:1009676403693)

[35] Froese R, Pauly D FishBase. See http://www.fishbase.org/search.php

[36] IUCN. 2013 The IUCN Red List of Threatened Species. Version 2013.2

[37] De La Vega-Salazar MY, Avila-Luna E, Macias-Garcia C 2003 Ecological evaluation of local extinction: the case of two genera of endemic Mexican fish, *Zoogoneticus* 12, 2043–2056. (doi:10.1023/a:1024155731112)

[38] Dominguez-Dominguez O, Zambrano L, Escalera-Vazquez LH, Perez-Rodriguez R, de Leon GPP 2008 Changes in the distribution of goodeids (Osteichthyes: Cyprinodontiformes: Goodeidae) in river basins of Central Mexico. *Revista Mexicana De Biodiversidad* 79, 501–512.

[39] Magurran AE 2009 Threats to freshwater fish. *Science* 325, 1215–1216. (doi:10.1126/science.1177215) 19729647 10.1126/science.1177215

[40] Pitcher TJ, Magurran AE, Winfield IJ 1982 Fish in larger shoals find food faster. *Behav. Ecol. Sociobiol.* 10, 149–151. (doi:10.1007/bf00300175)

[41] Valero A, Macías Garcia C, Magurran AE 2008 Heterospecific harassment of native endangered fishes by invasive guppies in Mexico. *Biol. Lett.* 4, 149–152. (doi:10.1098/rsbl.2007.0604) 18211863 10.1098/rsbl.2007.0604 PMC2429932

[42] Sievers C, Willing EM, Hoffmann M, Dreyer C, Ramnarine I, Magurran A 2012 Reasons for the invasive success of a guppy (Poecilia reticulata) population in Trinidad. *PLoS ONE* 7, 38404 (doi:10.1371/journal.pone.0038404) 10.1371/journal.pone.0038404 PMC3365015 22693621

[43] Griffiths SW, Magurran AE 1997 Familiarity in schooling fish: how long does it take to acquire??. *Anim. Behav.* 53, 945–949. (doi:10.1006/anbe.1996.0315)

[44] Macias-Garcia C 2013 Mode of reproduction, mate choice, and species richness in goodeid fish. In *Sexual selection, perspectives and models from the Neotropics* (eds Macedo R, Machado G), pp. 253–288. Amsterdam, The Netherlands: Elsevier.

[45] Hurlbert SH 1984 Pseudoreplication and the design of ecological field experiments. *Ecol. Monogr.* 54, 187–211. (doi:10.2307/1942661)

[46] R-Core-Team. 2013 *R: a language and environment for statistical computing.* Vienna, Austria: R Foundation for Statistical Computing.

[47] Laland KN, Williams K 1997 Shoaling generates social learning of foraging information in guppies. *Anim. Behav.* 53, 1161–1169. (doi:10.1006/anbe.1996.0318) 9236013 10.1006/anbe.1996.0318

[48] Reader SM, Kendal JR, Laland KN 2003 Social learning of foraging sites and escape routes in wild Trinidadian guppies. *Anim. Behav.* 66, 729–739. (doi:10.1006/anbe.2003.2252)

[49] Suboski MD, Templeton JJ 1989 Life skills training for hatchery fish—social-learning and survival. *Fish. Res.* 7, 343–352. (doi:10.1016/0165-7836(89)90066-0)

[50] Magurran AE, Higham A 1988 Information transfer across fish shoals under predation threat. *Ethology* 78, 153–158. (doi:10.1111/j.1439-0310.1988.tb00226.x)

[51] Cresswell W, Quinn JL, Whittingham MJ, Butler S 2003 Good foragers can also be good at detecting predators. *Proc. R. Soc. Lond. B* 270, 1069–1076. (doi:10.1098/rspb.2003.2353) 10.1098/rspb.2003.2353 PMC1691342 12803897

[52] Metcalfe NB 1989 Flocking preferences in relation to vigilance benefits and aggression costs in mixed-species shorebird flocks. *Oikos* 56, 91–98. (doi:10.2307/3566091)

[53] Lind J, Cresswell W 2005 Determining the fitness consequences of antipredation behavior. *Behav. Ecol.* 16, 945–956. (doi:10.1093/beheco/ari075)

[54] Lodge DM 1993 Biological invasions: lessons for ecology. *Trends Ecol. Evol.* 8, 133–137. (doi:10.1016/0169-5347(93)90025-k) 21236129 10.1016/0169-5347(93)90025-K

[55] Kolar CS, Lodge DM 2001 Progress in invasion biology: predicting invaders. *Trends Ecol. Evol.* 16, 199–204. (doi:10.1016/s0169-5347(01)02101-2) 11245943 10.1016/s0169-5347(01)02101-2

[56] Carvalho GR, Shaw PW, Hauser L, Seghers BH, Magurran AE 1996 Artificial introductions, evolutionary change and population differentiation in Trinidadian guppies (Poecilia reticulata: Poeciliidae). *Biol. J. Linnean Soc.* 57, 219–234. (doi:10.1111/j.1095-8312.1996.tb00310.x)

[57] Auer SK 2010 Phenotypic plasticity in adult life-history strategies compensates for a poor start in life in Trinidadian guppies (Poecilia reticulata). *Am. Nat.* 176, 818–829. (doi:10.1086/657061) 20973669 10.1086/657061

[58] Rodd FH, Reznick DN 1997 Variation in demography of guppy populations: the importance of predation and life histories. *Ecology* 78, 405–418. (doi:10.1890/0012-9658(1997)078[0405:VITDOG]2.0.CO;2)

[59] Garcia-Berthou E 2007 The characteristics of invasive fishes: what has been learned so far??. *J. Fish Biol.* 71, 33–55. (doi:10.1111/j.1095-8649.2007.01668.x)

[60] Simberloff D et al 2013 Impacts of biological invasions: what's what and the way forward. *Trends Ecol. Evol.* 28, 10 (doi:1016/j.tree.2012.07.013) 10.1016/j.tree.2012.07.013 22889499

